# Prevalence of the oral pathogen *Filifactor alocis* and its FtxA toxin related to clinical parameters and presence of *Aggregatibacter actinomycetemcomitans*

**DOI:** 10.3389/fcimb.2024.1501028

**Published:** 2025-01-22

**Authors:** Zeinab Razooqi, Nabil Khzam, Mahina L’Hostis, Georgios N. Belibasakis, Anders Johansson, Jan Oscarsson

**Affiliations:** ^1^ Department of Odontology, Umeå University, Umea, Sweden; ^2^ Oral Health Centre of Western Australia, Dental School, The University of Western Australia, Perth, WA, Australia; ^3^ NK Periodontics, Perth, WA, Australia; ^4^ Division of Oral Health and Periodontology, Department of Dental Medicine, Karolinska Institutet, Stockholm, Sweden

**Keywords:** *Filifactor alocis*, *ftxA*, RTX toxin, *Aggregatibacter actinomycetemcomitans*, periodontitis, clinical attachment loss (CAL), clinical parameters

## Abstract

The Gram-positive organism *Filifactor alocis* is implicated in multiple oral diseases including periodontitis, and approximately 50% of known strains encode and produce a recently identified repeat-in-toxin (RTX) protein, FtxA, partly homologous to the *Aggregatibacter actinomycetemcomitans* leukotoxin. By assessing a longitudinal Ghanaian study population of adolescents, we recently identified a possible correlation between *F. alocis* levels, *ftxA* gene carriage, and progression of clinical attachment loss (CAL). To extend knowledge on the possible significance of *F. alocis* and its FtxA in periodontal disease, we have in the present work analyzed saliva samples in an independent cohort of periodontitis (n=156), collected at two private periodontal specialist practices in Perth, Western Australia. The present results corroborate that high loads of *F. alocis* and the presence of its *ftxA* gene together are associated with parameters of periodontal tissue destruction and severity. Moreover, among the individuals carrying *A. actinomycetemcomitans*, a majority also exhibited an *ftxA*-positive *F. alocis*, supporting the notion of the synergistic behavior of these two species. This emphasizes that *F. alocis* and its *ftxA* are involved in the pathogenesis of periodontitis and may have ecological roles, with diagnostic and prognostic implications for the disease.

## 1 Introduction

Periodontitis is a bacterially induced oral inflammatory disorder, which eventually causes the degradation of periodontal tissues and tooth loss (Pihlstrom et al., 2005). It is a major oral health issue in public health, with high costs to society, ranking as the sixth most common infectious disease globally, and in the United States, 42% of adults over 30 have a form of periodontitis (Eke et al., 2018). The collective research to date supports that in scenarios where the overall balance among the resident periodontal polymicrobial bacterial communities is disturbed, a dysbiotic environment will result, and some of the species might exhibit potential pathogenic characteristics, leading to an inflammatory response in the periodontal tissues, which together with factors related to the host susceptibility promotes the degradation (Darveau, 2010; Hajishengallis and Lamont, 2021; Lamont et al., 2023). Typically, periodontitis affecting adolescents and young individuals has been referred to as aggressive periodontitis and is at present defined as stage IV and grade C, indicating rapid progress (Tonetti et al., 2018). There is a wide geographical spread with an enhanced incidence in North and West Africa (Bouziane et al., 2020; Yoshida et al., 2021; Kissa et al., 2022). Leukotoxin (LtxA) belongs to the repeats-in-toxin (RTX) family of toxins and is a major virulence factor of the Gram-negative species *Aggregatibacter actinomycetemcomitans*. As far as known, LtxA is produced by all hitherto recognized wild-type strains (Fine et al., 2020). A highly leukotoxin-producing genotype (JP2) is associated with the periodontal disease progress in these geographical regions in Africa (Haubek et al., 2008; Höglund Åberg et al., 2014). The JP2 genotype strains of this organism carry a deletion of 530 base pairs (bp) in the *ltxCABD* promoter (Brogan et al., 1994), which could be responsible for the enhanced production of leukotoxin in these strains by containing a potential transcriptional terminator sequence (Sampathkumar et al., 2017). To the best of our knowledge, few studies executed in the northern and western geographical regions of Africa have been reported regarding the relation between periodontitis and the progression of this disease in young individuals, and concomitantly focusing on bacterial species other than *A. actinomycetemcomitans* (Dahlén et al., 2014; Yoshida et al., 2021), thus including the Gram-positive, anaerobic organism *Filifactor alocis*.

*F. alocis* has recently been identified in the oral microbiome via metagenome DNA sequencing. It is culturable in the laboratory and is considered an emerging oral pathogen implicated in the etiology of periodontal (Aruni et al., 2014; Greenwood et al., 2020), peri-implantitis (Sanz-Martin et al., 2017), and endodontic (Zehnder et al., 2017) infections. It is a potential biomarker for active disease in young children (Aruni et al., 2014). *F. alocis* seems to have a synergistic relationship with *A. actinomycetemcomitans* in periodontitis (Fine et al., 2013; Wang et al., 2013). However, *A. actinomycetemcomitans* appears thereafter to be outcompeted by *F. alocis* in the deeper pockets (Dahlén et al., 2014). This suggests that *F. alocis* is able to adapt and drive the disease process forward in the absence of *A. actinomycetemcomitans* via as yet unknown mechanisms.

Approximately 50% of isolated strains of *F. alocis* encode a recently identified RTX protein, FtxA (Oscarsson et al., 2020; Bao et al., 2022; Ozuna et al., 2022). We recently assessed 2-year longitudinal data from a study population of adolescents in Ghana (Höglund Åberg et al., 2012; Höglund Åberg et al., 2014). This first revealed a possible correlation between deep periodontal pockets measured at the 2-year follow-up, the presence of the *ftxA* gene, and a high quantity of *F. alocis* (Razooqi et al., 2022). To further understand the contribution of *F. alocis* and FtxA in periodontal disease, we then quantified the carriage loads of *F. alocis* and the prevalence of its *ftxA* gene in subgingival plaque specimens, sampled at baseline from this Ghanaian cohort (Razooqi et al., 2024). Comparing these results with the recorded clinical attachment loss (CAL) longitudinal progression data from the 2-year follow up, we concluded that carriers of *ftxA*-positive *F. alocis* typically exhibited higher loads of the bacterium. Moreover, high carriage loads of *F. alocis* and the concomitant presence of the *ftxA* gene were two factors that were associated with an enhanced prevalence of CAL progression. Interestingly, CAL progression appeared to be further promoted upon the simultaneous presence of *F. alocis* and the non-JP2 genotype of *A. actinomycetemcomitans* (Razooqi et al., 2024). These results hence were consistent with the notion that *F. alocis* and its *ftxA* gene promotes CAL during periodontal disease.

Our previous work focused on adolescents while here we wanted to confirm the possible significance of *F. alocis* and its FtxA in association with periodontal disease in a wider age range, in a different study population, and in different disease severities. Hence, we have in the present work assessed saliva samples from a collection of patients all diagnosed with periodontitis (n=156; ages 26-86), collected at two private periodontal specialist practices in Perth, Western Australia. Here, we report on correlations between the presence and loads of *F. alocis* and its *ftxA* with clinical and demographic parameters, and with the presence of *A. actinomycetemcomitans*.

## 2 Materials and methods

### 2.1 Study population

**Figure 1:**
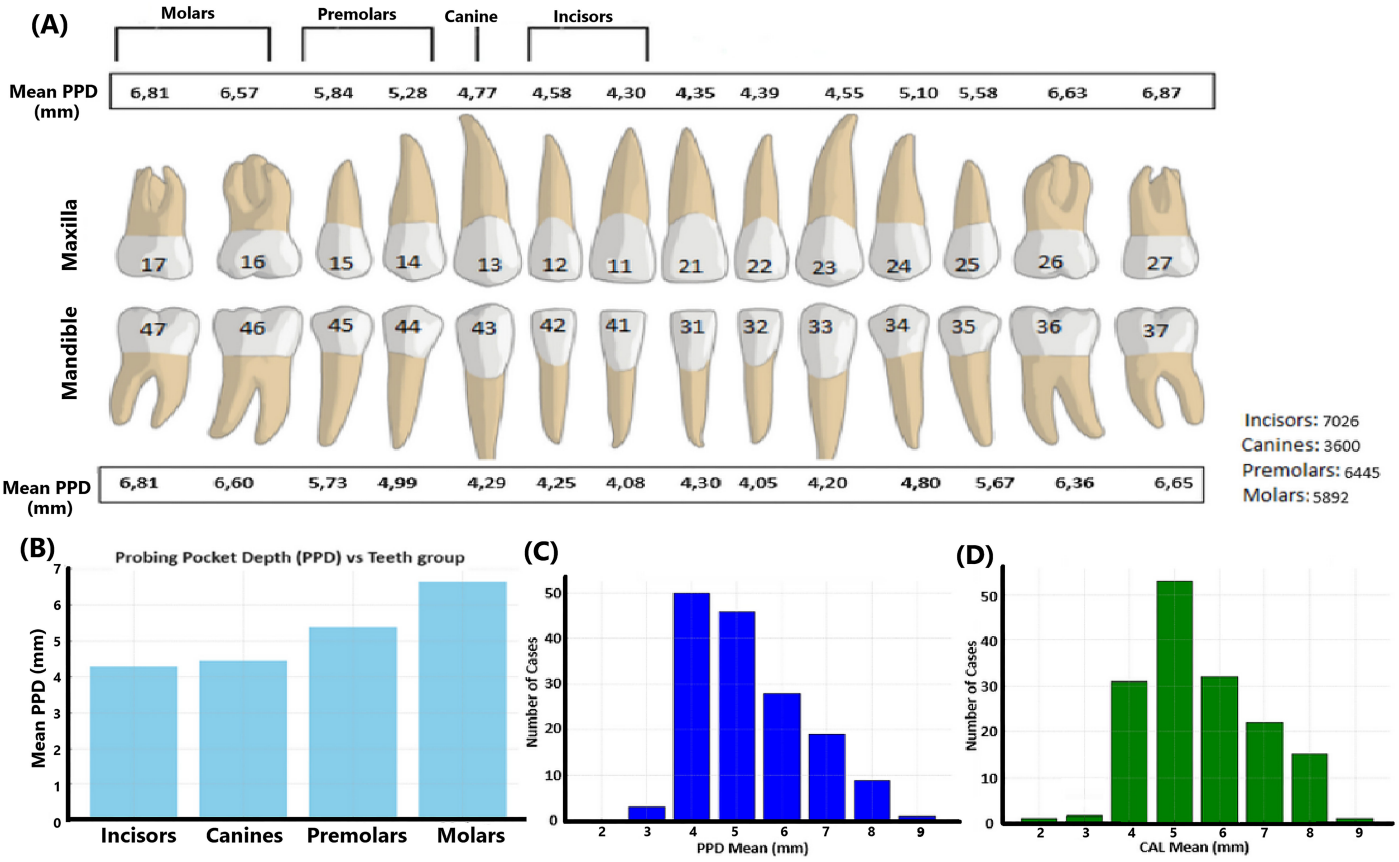
Mean periodontal pocket depth data of the present study population. **(A)** Illustration of the PPD measurements across the dentition in millimeters. The maxillary and mandibular teeth are represented with corresponding mean PPD values for each tooth type, including incisors, canines, premolars, and molars. The total number of teeth in each category is also provided, indicating the distribution of the cases studied. **(B)** Mean PPD values across the four groups of teeth: incisors, canines, premolars, and molars. **(C)** Distribution of cases based on the PPD mean values based on all dental sites assessed in each patient. **(D)** Distribution of cases based on the CAL mean values based on all dental sites assessed in each patient. Numbers on teeth are according to the documentation used in dentistry in Sweden.

All the clinical specimens used in the present work are salivary samples from a population of periodontally diseased patients (n=156; ages 26-86; 73 men, and 83 women) in Perth, Western Australia (Khzam et al., 2024). Extensive clinical parameters and demographic data on this population have been described (Khzam et al., 2024). The graphical representation of the means of periodontal pocket depth (PPD) across tooth groups in this population demonstrates a clear trend where molars exhibit the highest PPD (**Figure 1**).

### 2.2 DNA extraction

The saliva samples were subject to genomic DNA isolation using a GXT NA Extraction Kit^®^ (Hain Lifesience, GmBH, Nehren, Germany), and an Arrow extraction instrument (Liaison IXT, DiaSorin Ltd., Fort Henry, Ireland) using procedures described previously (Razooqi et al., 2022). In brief, for pre-treatment, 400 μl of each saliva sample was mixed with 400 μl lysis buffer. To the mixture 20 μl proteinase K (20 mg/ml) was added, and then the samples were incubated at 55°C for 30 min. The DNA was extracted from 550 μl of the mix, eluted in a volume of 100 μl, and stored at 4°C prior to use.

### 2.3 Quantitative PCR analysis

For this, DNA samples from saliva were used as templates in qPCR for each of the individuals (n=156). The KAPA SYBR^®^ FAST qPCR Kit (Kapa Biosystems, Wilmington, MA, USA) was used, with cycling conditions as described previously (Razooqi et al., 2022), and with a forward (5’-CAGGTGGTTTAACAAGTTAGTGG-3’), and a reverse (5’-CTAAGTTGTCCTTAGCTGTCTCG-3’) oligonucleotide primer, respectively, targeting the *F. alocis* 16s rRNA gene (Siqueira and Rocas, 2003). These primers have been found to be very efficient for the determination of *F. alocis* loads by qPCR in our previous work, and assays were standardized based on serially diluted samples of *F. alocis* cells (Razooqi et al., 2022, Razooqi et al., 2024). Loads of *F. alocis* were determined as numbers of cells/ml of the sample as described previously (Razooqi et al., 2022). Cut-off values of 10,000 and 100 F*. alocis* cells/ml of the sample were then used to divide the individuals as carriers of “high” (>10,000), and “low” (100<10,000) loads of *F. alocis* per sample, respectively. Loads <100 cells/sample were determined as negative for this bacterium. The presence of *A. actinomycetemcomitans* in the saliva samples was determined by qPCR, using a forward primer (5’-CTAGGTATTGCGAAACAATTTG-3’), and a reverse primer (5’-CCTGAAATTAAGCTGGTAATC-3’), and the cycling conditions were as described previously (Kirakodu et al., 2008). A load of 100 A*. actinomyctemcomitans* cells per ml of the sample was set as a positive result, in line with our recent work (Khzam et al., 2024).

### 2.4 PCR determination of presence of the *ftxA* gene of *F. alocis*

A forward (5′-GGCTCAGATACCTACTTCTTC-3′) and a reverse (5′-GAAGGCTATGATTTGATTGTTTCC-3′) oligonucleotide primer were used to amplify a 798-base pair (bp) internal fragment of the *ftxA* gene, as described previously (Oscarsson et al., 2020; Razooqi et al., 2022). Results were scored as positive or negative for *ftxA* when this amplicon was obtained or not, as visualized on agarose gels.

### 2.5 Statistical analysis

Data analyses were performed using the Chi-square and Fisher’s exact tests, using SPSS 22.0 (SPSS Inc., Chicago, IL, USA) or Microsoft Excel (version 16.80). Input microbial data were (i) presence, and levels of *F. alocis* (*i.e.*, present/absent, and high, low, or negative, respectively), (ii) presence of *ftxA* (yes or no), and (iii) presence of *A. actinomycetemcomitans* (yes or no), determined as described above. Microbial data were initially assessed for associations between *F. alocis* and/or *ftxA*, and *A. actinomycetemcomitans*, and then with the respective clinical parameters. These are summarized in **Table 1**. Significant differences between sample groups were examined using the Mann–Whitney U test or *t*-test. Results were estimated by an odds ratio (OR) with a 95% confidence interval (CI).

**Table 1:** Microbial data and clinical parameters of the study population (n=156) assessed in the present work.

Clinical parameter^1^	Scored readout
Gender (83 women, 73 men)	Woman, man
Age (age span in population 26-86y)	≤40 y, >40 y
Extent	Local/General periodontitis
Bone loss/age	<0.25, 0.25-1, >1
Grade of periodontal disease	A, B, C
Stage of periodontal disease	I, II, III, IV
Periodontal pocket depth (PPD)	Means (mm)
Clinical attachment loss (CAL)	Means (mm)
History of periodontal disease in family	Yes, no

^1^Determined earlier (Khzam et al., 2024).

### 2.6 Ethics statement

The studies in the present work were approved by the ethical committee of the University of Western Australia (Ref no. 2022/ET000252; June 13, 2022; and updated April 5, 2023), and by the Swedish Ethical Review Authority (dnr 2024-01704-01).

## 3 Results

### 3.1 Carriers of *ftxA* ^+^ *F. alocis* exhibit higher loads of this bacterium

**Figure 2:**
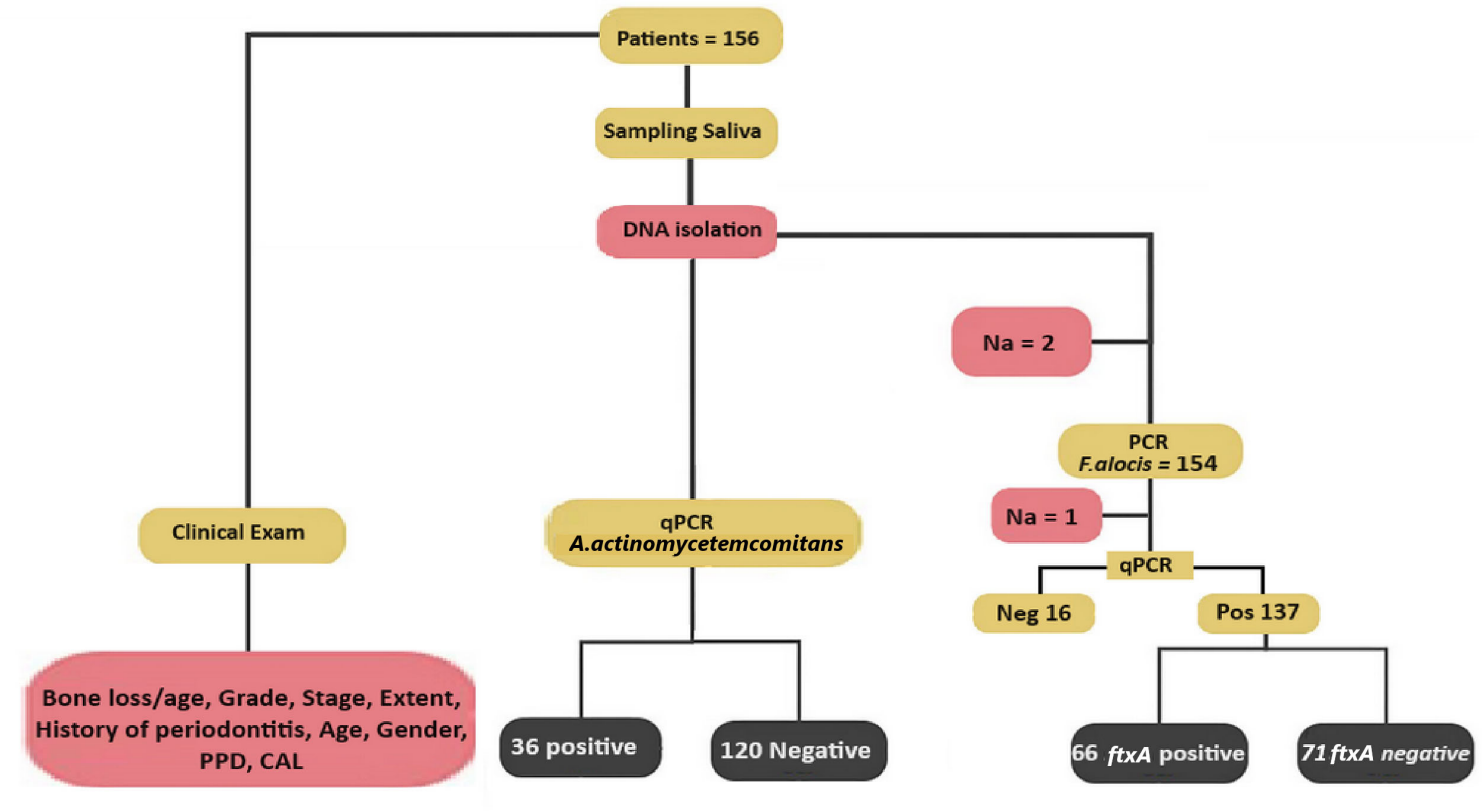
Scheme illustrating the design of the present study. Multiple clinical parameters from each of the 156 patients were recorded previously (Khzam et al., 2024). In the saliva samples, *F. alocis* levels were determined by qPCR, and the samples considered positive for *F. alocis* were genotyped for the presence or absence of the *ftxA* gene using PCR. The presence of *A. actinomyetemcomitans* in the samples was also set as positive or negative, deduced via qPCR. Also indicated is the number of non-analyzed samples (Na), which represent samples that were not analyzed due to a lack of material.

**Figure 3:**
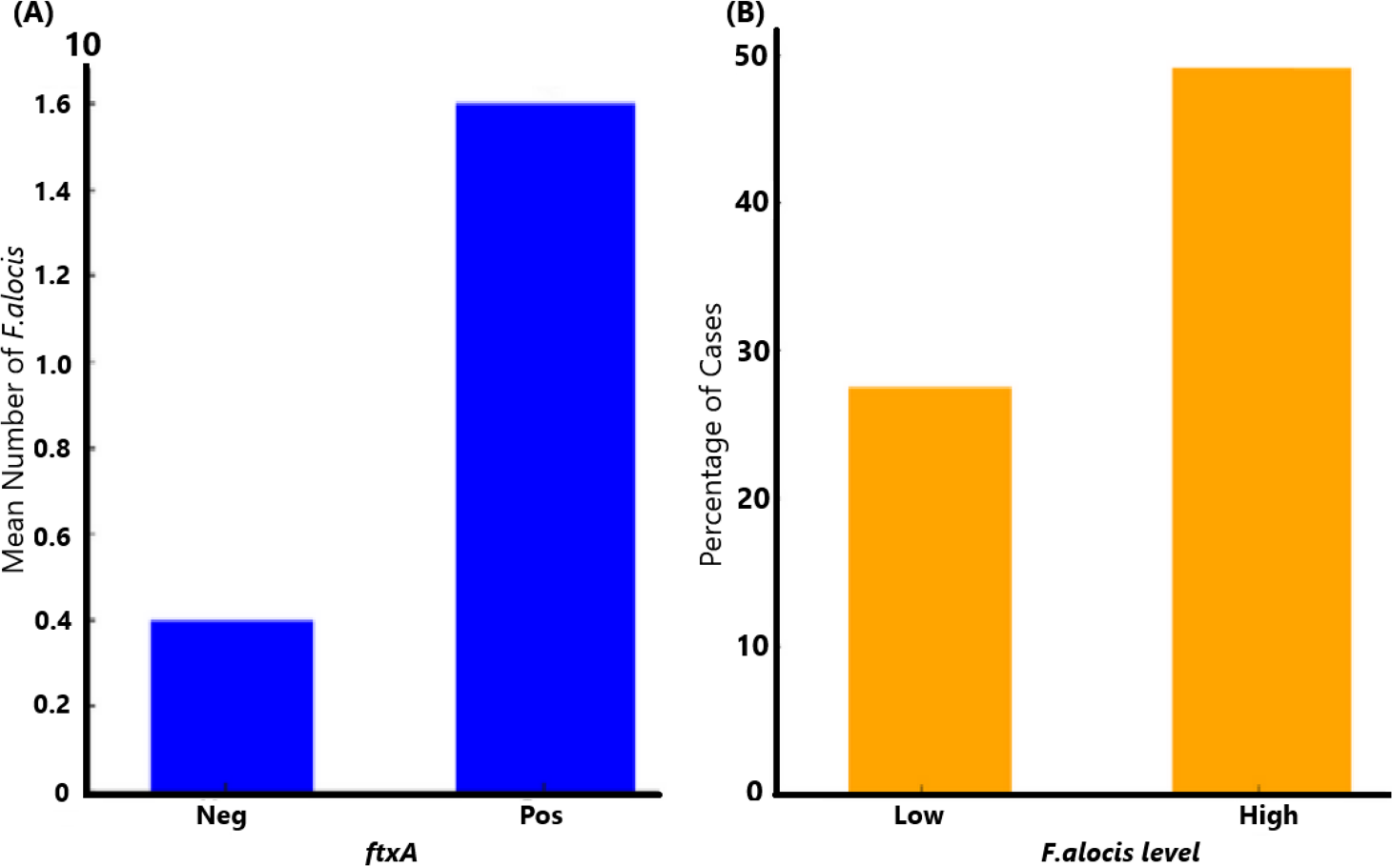
Association of *ftxA* with high loads of *F. alocis* among carriers. **(A)** Comparison of mean *F. alocis* (FA) counts (cells per sample) between *ftxA*-negative (Neg), and positive (Pos) cases (*p*=0.001). **(B)** High levels of *F. alocis* were a property of a majority of the carriers of an *ftxA*-positive *F. alocis* (*p*=0.018).

A flow chart summarizing the outline of the present study is shown in **Figure 2**. The qPCR analysis revealed that 137 of 154 (89.0%) assessed patients carried *F. alocis* (*i.e.*, exhibited a load of >100 F*. alocis* per sample). Among these *F. alocis*-positive patients, 113 (82.5%) exhibited high levels of this bacterium (>10,000 per sample). Moreover, of the 153 assessed patients, 66 (43.1%) were found to be *ftxA*-positive using PCR. Interestingly, a majority of these 66 *ftxA*-positive individuals, 55 (83.3%), had high loads (>10,000 cells of *F. alocis* per sample; **Figure 3**). This supports the idea that *ftxA* is associated with high loads of *F. alocis* among carriers, and that high levels of *F. alocis* is a property of a majority of the carriers of an *ftxA*-positive *F. alocis*.

### 3.2 Co-existence of *A. actinomycetemcomitans* with *F. alocis* and *ftxA* in the study population

**Figure 4:**
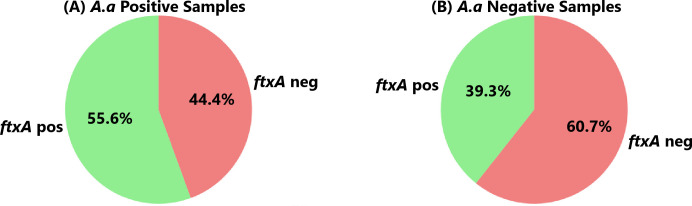
Co-carriage of an *ftxA*-positive *F. alocis* in **(A)** the 36 individuals who carried *A. actinomycetemcomitans* (*A.a*. Positive), and in **(B)** the 136 who lacked this bacterium (*A.a.* Negative), respectively. (*p*=0.085).

According to qPCR, *A. actinomycetemcomitans* was present in 36 (23.1%) of 156 analyzed samples (**Figure 2**). Of these 36 samples, 34 (94.4%) carried *F. alocis*, 28 (77.8%) had high loads of the bacterium (>10,000 cells/sample), and 20 (55.6%) were *ftxA*-positive (**Figure 4**).

*A. actinomycetemcomitans*-negative samples (n=120), however, exhibited a similar prevalence to *F. alocis* (104 were positive; 86.7%). Of these 104, 85 (70.8%) exhibited high loads of *F. alocis*, and 47 (39.3%) were *ftxA*-positive (**Figure 4B**). Thus, these results revealed no apparent trend regarding the presence of *F. alocis* in the samples regardless of whether *A. actinomycetyemcomitans* was present or not, albeit with a potential association with co-presence of *A. actinomycetemcomitans* with *ftxA*, in this periodontally diseased population.

### 3.3 *F. alocis* loads and *ftxA*-presence in relation to clinical parameters

These bacterial parameters were assessed in relation to the clinical parameters listed in **Table 1**.

#### 3.3.1 Bone loss/age

**Figure 5:**
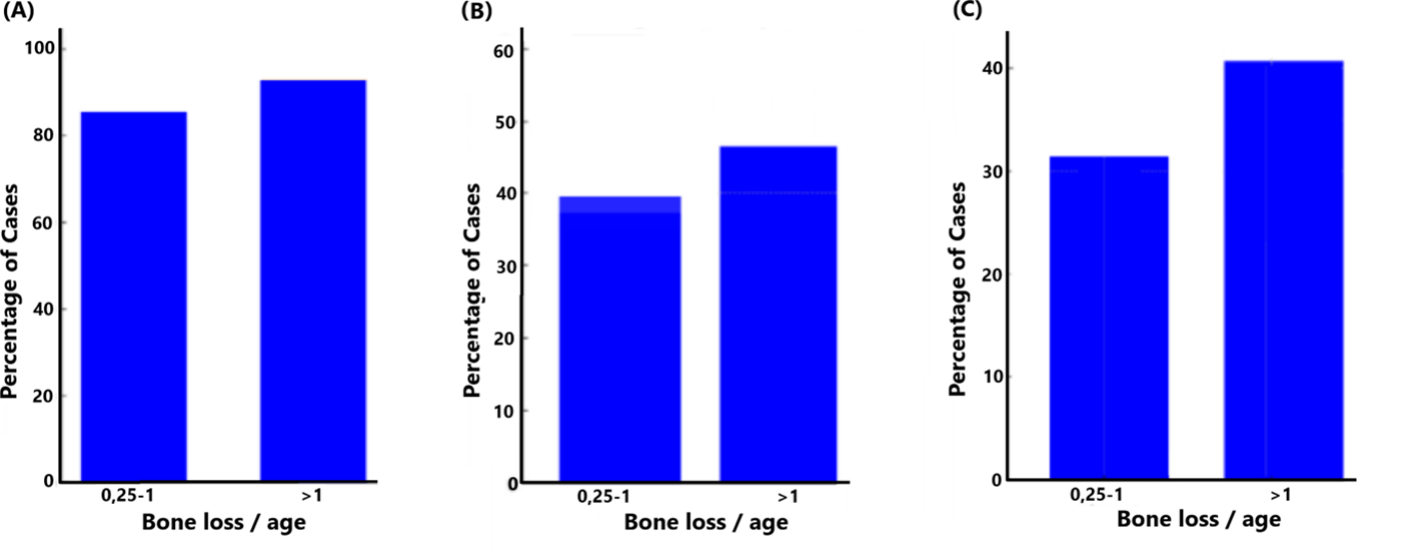
Relationship between bone loss/age and the presence of *F. alocis* (**A)**; *p*=0.177), *ftxA* (**B**; *p*=0.309), and combined high *F. alocis* loads with *ftxA* positivity (**C**; *p*=0.191).

It was observed that the proportion of *F. alocis*-positive cases was consistently high across both levels of bone loss (**Figure 5A**). Where bone loss was in the range of 0.25-1 or greater than 1, respectively, >80% of the cases were positive for *F. alocis*, suggesting a strong and persistent association between *F. alocis* presence and bone loss. Comparing the same bone loss parameters with the presence of *ftxA* revealed that 40% of the cases tested positive for *ftxA* when bone loss was in the range of 0.25-1 (**Figure 5B**). This percentage increased slightly when bone loss exceeded 1, with nearly 45% of cases being *ftxA* positive, suggesting a possible correlation between increased bone loss and *ftxA* positivity. Finally, these bone loss parameters were compared with simultaneous carriage of high levels of *F. alocis* and *ftxA* (**Figure 5C**). Approximately 30% of the cases with lower bone loss (0.25-1) were found to be positive for both high *F. alocis* loads and *ftxA*. The number of cases increased to approximately 40% when bone loss was greater than 1. This trend highlights a potential cumulative effect of high *F. alocis* levels and *ftxA* positivity in cases with severe bone loss, albeit with no significant positive association.

#### 3.3.2 Age group

**Figure 6:**
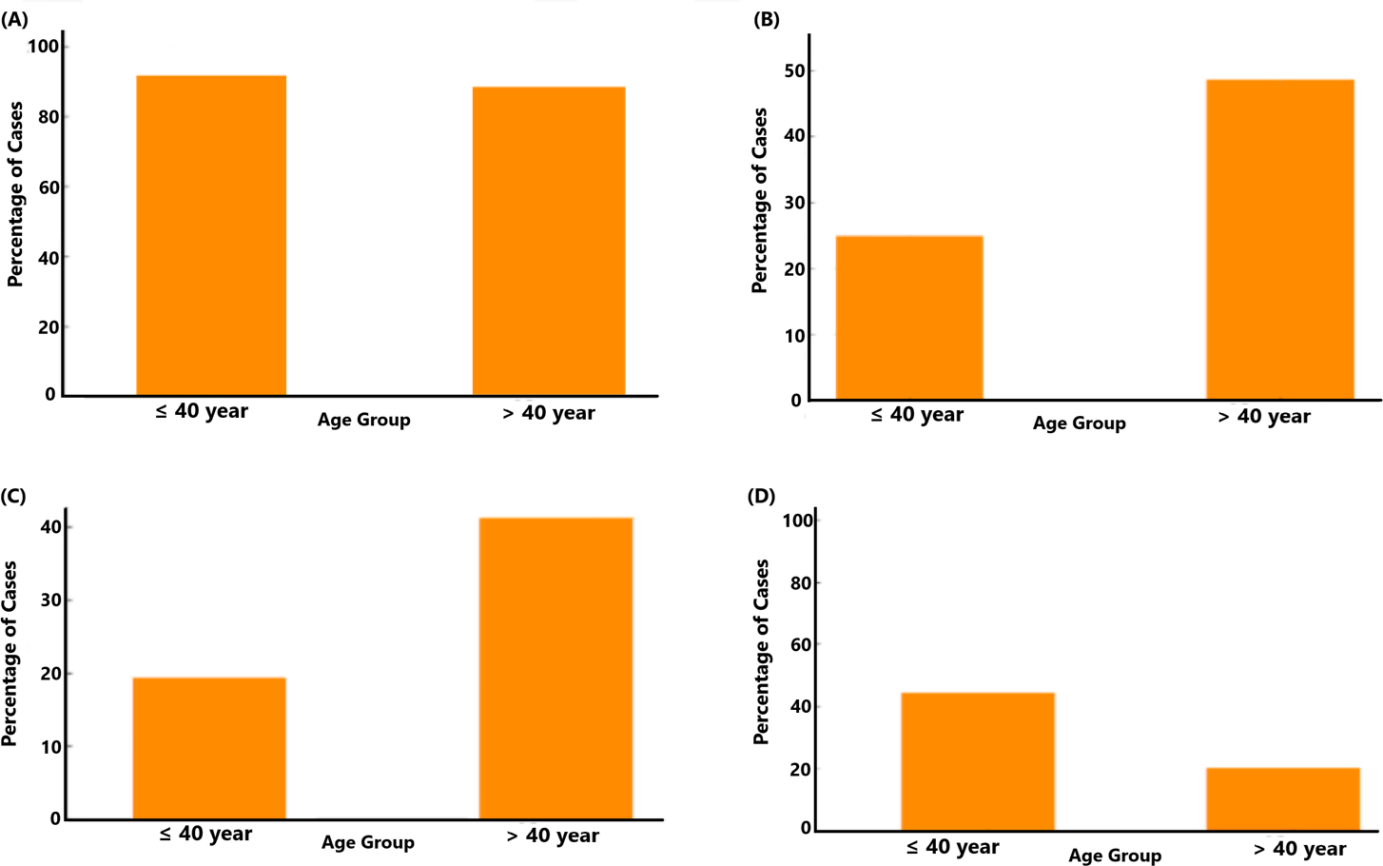
Relationship between age group (>40 years; n=36, and ≤40 years; n=117) and the presence of *F.* alocis (**A**; p=0.645), ftxA (**B**; p=0.012), combined high *F. alocis* loads and *ftxA* positivity (**C**; *p*=0.017), and *A. actinomycetemcomitans* (**D**; *p*=0.22).

The prevalence of *F. alocis* was assessed among two age groups, *i.e.*, individuals aged 40 years or younger and those older than 40 years, respectively. In both age groups, a majority of individuals tested positive for *F. alocis*, but a slightly higher percentage of the ≤40 years group tested positive for *F. alocis* (**Figure 6A**). This suggests a potential trend that age may not be a significant factor governing the prevalence of *F. alocis* within the population studied. However, assessing the distribution of *ftxA* across these two age groups (**Figure 6B**) revealed that, among individuals aged ≤40 years, approximately 25% tested *ftxA*-positive. A similar distribution was observed in the >40 years age group, with a clear increase in the proportion of *ftxA*-positive cases to approximately 50%. These results suggest a potential age-related trend in *ftxA*-positivity, with older individuals showing a higher frequency of carriage of *ftxA*-positive *F. alocis*. Assessing the distribution of individuals with high loads of *F. alocis* and simultaneous presence of *ftxA* across the two age groups (**Figure 6C**) revealed that among the ≤40 years, approximately 19% were identified as carriers of high *F. alocis* loads and *ftxA*-positivity. In contrast, the proportion of high *F. alocis* levels and co-presence of *ftxA* increased markedly in the group >40 years, with approximately 40%. This supports a clear age-related trend, where older individuals exhibit a notably higher percentage of high loads of *F. alocis* and a high simultaneous presence of *ftxA*, suggesting a potential increased susceptibility or exposure in this demographic. Finally, we addressed the distribution of *A. actinomycetemcomitans* between the two age groups (**Figure 6D**). For the ≤40 years, 43% of cases were positive for *A. actinomycetemcomitans*. In contrast, the percentage of carriers of this bacterium decreased significantly in the >40 years age group (approximately 19%). This suggests the possibility of a higher prevalence of carriage of *A. actinomycetemcomitans* among the younger individuals, and a potential age-related decline in the detection of this bacterium over time.

#### 3.3.3 Presence of *A. actinomycetemcomitans* by CAL

**Figure 7:**
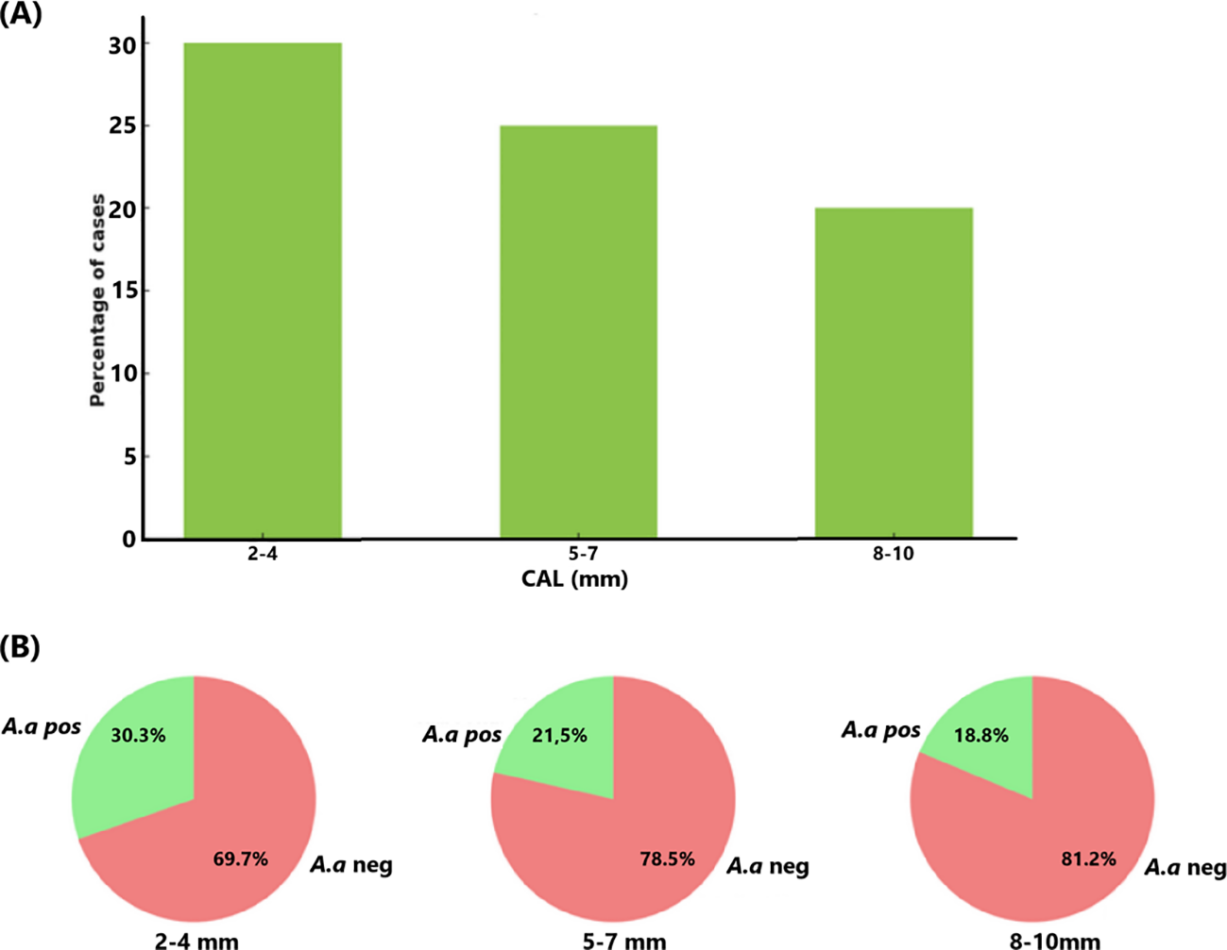
Relationship between CAL (mm) and the presence *of A. actinomycetemcomitans* (*A.a*) in the studied population (n=156) **(A)**. The proportion (%) of *A. actinomycetemcomitans*-positive cases reduces as CAL increases **(B)**.

In the present study population (n=156; 23.1% testing positive for *A. actinomycetemcomitans*), all subjects were assessed across different levels of CAL measured in millimeters (**Figure 7**). The results indicate that upon increasing CAL, the presence of *A. actinomycetemcomitans* decreased. Approximately 30% of the individuals with a CAL of 2-4 mm tested positive for *A. actinomycetemcomitans*. This frequency was lower, *i.e.*, ≈22% for the individuals with a CAL of 5-7. This trend continued with 18% of *A. actinomycetemcomitans*-positive individuals exhibiting a CAL of 8-10 mm. These data together suggest that *A. actinomycetemcomitans* is more common in individuals with less attachment loss, reflecting a potential trend that this organism may be less present in deeper periodontal pockets.

#### 3.3.4 Grade and stage of periodontitis

**Figure 8:**
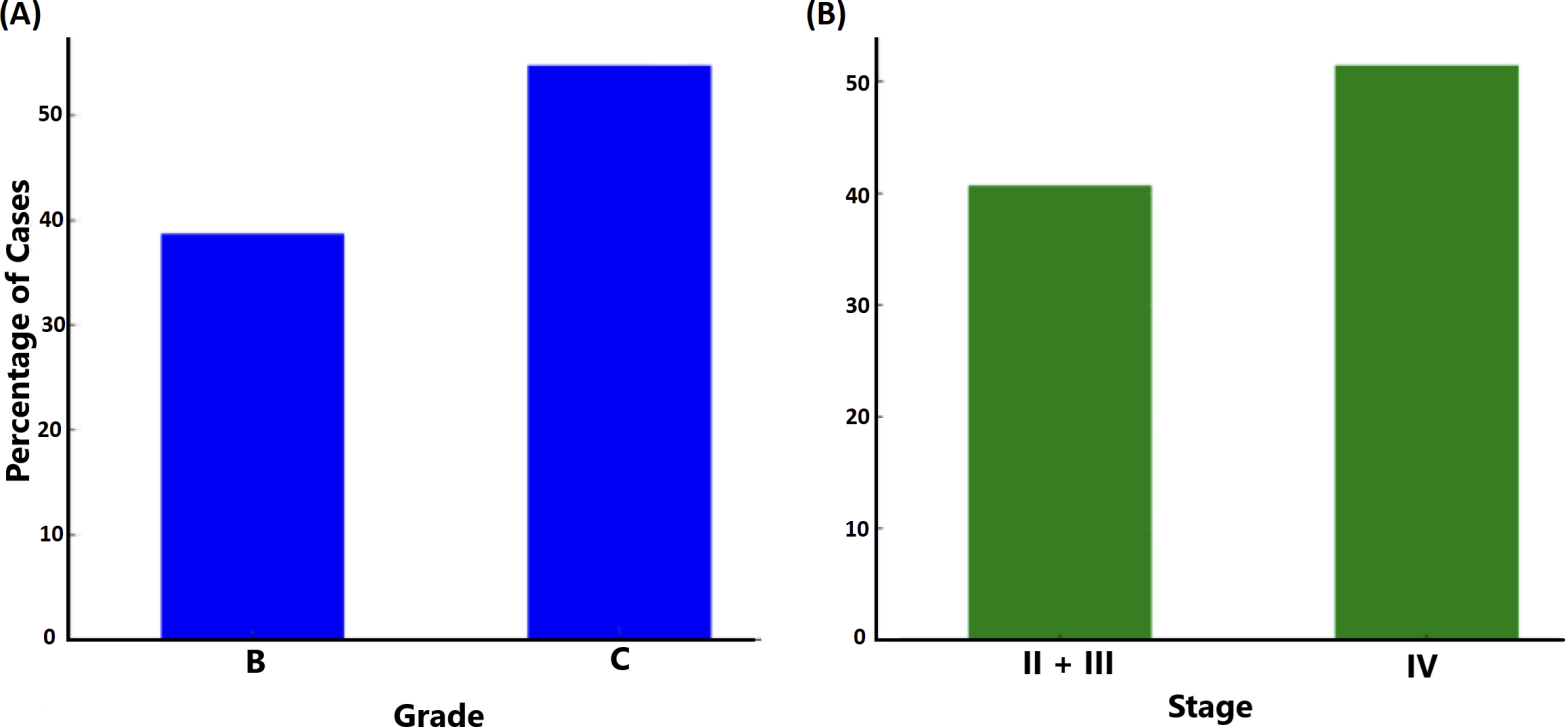
Relationship between grade (**A**; *p*=0.075) and stage (**B**; *p*=0.259) of periodontal disease and carriage of *ftxA* (% of carriers).

Grade and stage were classified according to the established classification criteria (Kornman and Papapanou, 2020). Assessing *ftxA*-positivity among the 156 samples in relation to disease grade (**Figure 8A**) revealed that the percentage of *ftxA*-positive cases increased from approximately 39% in Grade B (representing 43 cases of 111) to ≈53% (23 cases of 42) in Grade C. This indicates an increase in *ftxA*-positivity for Grade C, suggesting a potential correlation between higher grades and *ftxA* presence. A similar potential trend was observed when monitoring carriage of *ftxA* across different stages of the disease, with stages II and III combined, and compared against stage IV (**Figure 8B**). The frequency of *ftxA*-positive carriers was approximately 40% (48 cases) in the combined stages II and III, and this percentage rose to nearly 51% (18 of 35 cases) in stage IV. This indicates an increase in *ftxA*-positivity in more advanced stages of the disease, indicating that the presence of this RTX protein may be more prevalent as the disease progresses to later stages.

#### 3.3.5 Extent of periodontitis (local and generalized)

**Figure 9:**
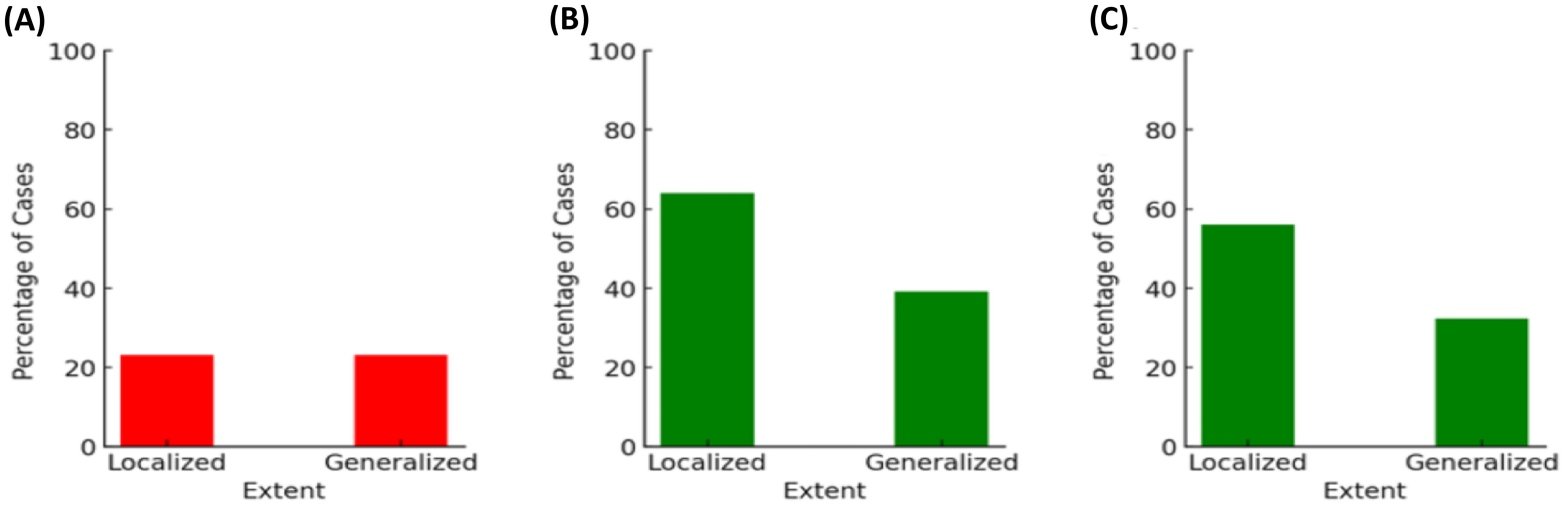
The extent of periodontitis analyzed regarding possible associations with the presence of *A. actinomycetemcomitans* (**A**; *p*=1.00), *ftxA* (**B**; *p*=0.027), and high loads of *F. alocis* in combination with the presence of *ftxA* (**C**; *p*=0.025), respectively. The percentages of cases (%) are on the basis of the 156 assessed individuals for *A. actinomycetemcomitans*, and 153 for *F. alocis* loads and *ftxA*, respectively.

The extent of periodontitis was assessed regarding possible associations with the presence of *A. actinomycetemcomitans*, *ftxA*, and with high loads of *F. alocis* in combination with the presence of *ftxA*, respectively. As shown in **Figure 9A**, the distribution of *A. actinomycetemcomitans*-positive cases showed no difference between localized and generalized forms of periodontitis. This indicates no association between the presence of *A. actinomycetemcomitans* and the form of periodontal disease. Moreover, *ftxA* positivity was more frequently observed in localized forms of the disease. Assessment of the extent of periodontitis and presence of *ftxA* (**Figure 9B**) revealed a higher percentage of *ftxA*-positive cases with localized periodontitis (≈64%; 16 cases), compared to those with generalized periodontitis (39%; 50 *ftxA*-positive cases). Finally, cases with both high loads of *F. alocis* and *ftxA* were concluded to be more prevalent when diagnosed with localized periodontitis (56%, 14 cases) compared to generalized periodontitis (32%, 41 cases; **Figure 9C**). Thus, taken together, these results indicate that *F. alocis* and its *ftxA* can play a role in the extent of periodontitis.

#### 3.3.6 History of periodontitis

**Figure 10:**
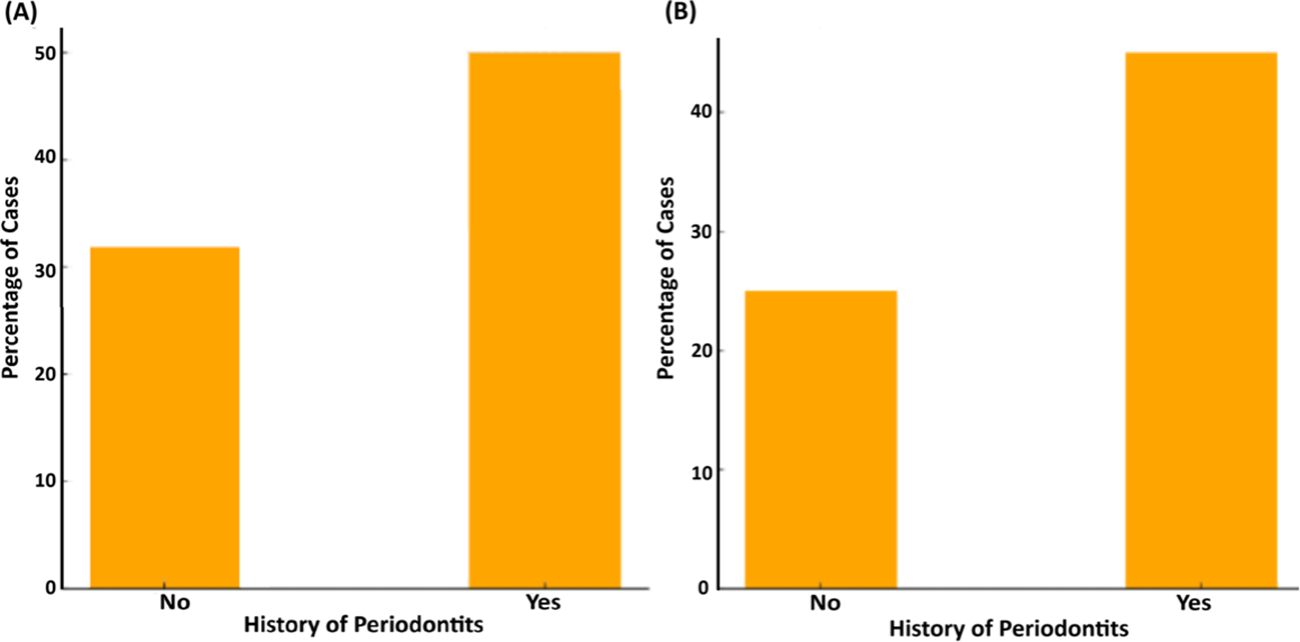
Association between history of periodontitis in the family and the presence of *ftxA* (**A**; *p*=0.061), and the combined presence of high *F. alocis* loads and *ftxA* (**B**; *p*=0.016). Frequency of cases is displayed as a percentage (%) of the total number of assessed cases (n=153).

Our present data indicate that individuals with a history of periodontal disease in the family exhibited a higher prevalence of *ftxA*-positivity, representing nearly 50% (n=42) of the cases (**Figure 10**). In contrast, only ≈35% (20 cases) of individuals without a history of periodontitis were found to be *ftxA*-positive. This suggests a strong link between a history of periodontitis in the family and carriage of an *ftxA*-positive *F. alocis*. Moreover, ≈45% (37 cases) of the individuals with a family history of periodontitis appeared to exhibit both high levels of *F. alocis* and the presence of *ftxA*, compared to those without a history of periodontitis (25%, 14 cases). This suggests a strong link between a family history of periodontitis and the presence of *ftxA*, and/or simultaneous carriage of high loads of *F. alocis* and the presence of *ftxA*.

#### 3.3.7 Gender

**Figure 11:**
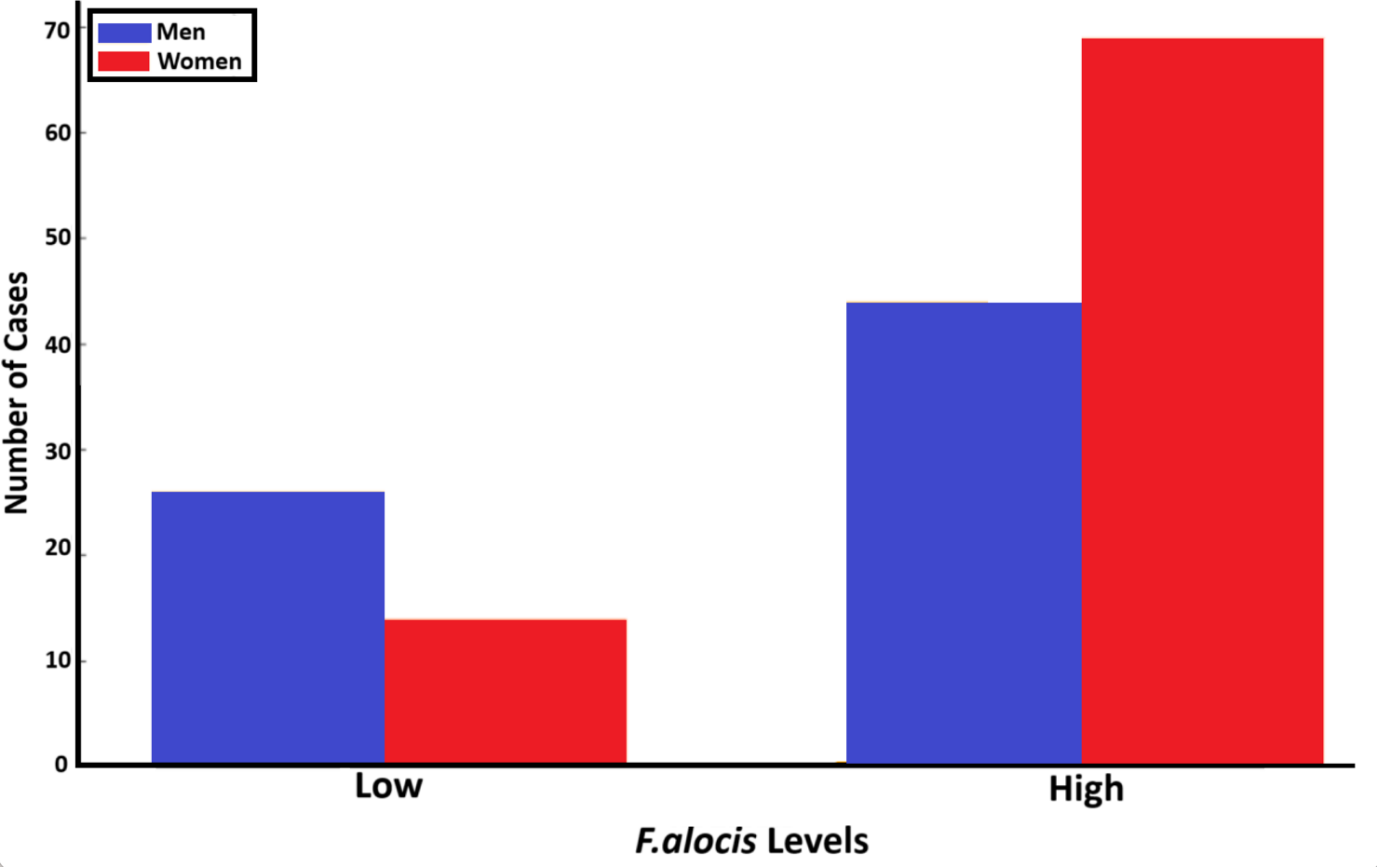
Relationship between the gender of individuals in the studied population (n=153) and levels of *F. alocis* carried (low and high, respectively) (*p*=0.011).

Finally, we addressed the loads of *F. alocis* (high versus low) related to gender in the sampled population (n=153; **Figure 11**). This revealed that for the carriers of low *F. alocis* levels, men represented a higher proportion of cases (26; 65%), compared to women (14 cases; 35%). Among the carriers of high *F. alocis* loads, women represented a higher proportion with 69 cases (61%) compared to men (44; 39%). These results highlight potential gender-related differences related to the levels of *F. alocis* within the studied population.

## 4 Discussion

In the present work, we used qPCR and PCR to characterize the prevalence and carriage loads of the emerging oral pathogen *F. alocis*, and its RTX-toxin-encoding gene *ftxA*, respectively, in salivary specimens previously sampled from 156 patients (aged 26-86 years), all diagnosed with periodontitis (Khzam et al., 2024). The obtained results were thereafter compared with our qPCR data on the presence or absence of *A. actinomycetemcomitans*, and earlier recorded clinical and demographic data for eight parameters (including age, bone loss/age, extent, grade and stage of periodontitis, PPD, and CAL) from these individuals (Khzam et al., 2024).

Despite periodontitis being a complex multifactorial disease, involving multiple host factors and complex microbiota, our focus was here, in the present approach, essentially on a single bacterial organism, *F. alocis*, to expand our knowledge of the virulence characteristics of individual bacteria linked to clinical parameters. *F. alocis* has attracted further interest recently after the discovery of genotypes that express FtxA, via its gene *ftxA*, which is the hitherto second identified RTX toxin in the oral cavity after the well-studied leukotoxin of the periodontopathogen *A. actinomycetemcomitans* (Johansson, 2011; Oscarsson et al., 2020; Bao et al., 2022; Ozuna et al., 2022). The functional mechanism(s) of FtxA are not yet known. However, interestingly, a potential role of FtxA in the virulence of *F. alocis* in periodontal disease was recently demonstrated upon assessment of the longitudinally followed Ghana adolescent cohort, revealing that carriers of *ftxA*-positive *F. alocis* typically exhibited higher loads of the bacterium and also had an enhanced prevalence of CAL progression (Razooqi et al., 2022; Razooqi et al., 2024). The rationale for selecting a population with all individuals (n=156) diagnosed with periodontitis in the present work was its suitability in allowing further assessment of the role of *F. alocis* and its expressed FtxA in periodontal disease.

The presence of *F. alocis perse* in the samples appeared not to be clearly associated with any clinical or demographic parameter, which most likely was due to the high prevalence of this bacterium in the present study population (89%), which could mask any potential differences. However, consistent with our studies on the Ghanaian adolescent cohort (Razooqi et al., 2022; Razooqi et al., 2024), there was also a strong association in a recently tested West Australian population between *ftxA* positivity and elevated *F. alocis* levels. This suggests a potential role of FtxA in enhancing the pathogenicity of *F. alocis.* Whether this may be via enhancing the proliferation of *F. alocis* in the periodontal pocket and/or by augmenting its virulence via host cell modulation is not known. The correlation between *ftxA* positivity and enhanced bone loss, and moreover with the diagnosed extent of periodontitis (local and generalized), support the notion that presence of this RTX toxin gene increases the risk for more severe disease among carriers. As might be expected, the simultaneous presence of *F. alocis* at high levels and *ftxA* in samples revealed a cumulative effect as evidenced by a significantly greater disease severity, again as measured by bone loss and extent of periodontitis. These correlations suggest that *ftxA* could serve as a biomarker for identifying individuals at higher risk for rapid progression of periodontitis, highlighting its potential value in clinical diagnostics.

A potentially synergistic behavior between *F. alocis* and *A. actinocytetemcomitans* was initially implicated based on observations using the human oral microbe infection microarray (HOMIM), concluding that *A. actinomycetemcomitans*, *Streptococcus parasanguinis*, and *F. alocis* together may represent a consortium in the causation of localized aggressive periodontitis, by indicating sites of future bone loss (Fine et al., 2013). This notion has since then been further supported by us and others by analyzing the Ghanaian longitudinal adolescent cohort, reflecting that in a potential scenario in which these species are co-present in the periodontal pocket that *A. actinomycetemcomitans* may be successively out-competed by *F. alocis* (Dahlén et al., 2014; Razooqi et al., 2022; Razooqi et al., 2024). This was supported by our present observations of clinical readouts reflecting CAL. Moreover, in the present work, it was revealed that among the individuals carrying *A. actinomycetemcomitans*, a majority also exhibited an *ftxA*-positive *F. alocis*. Regarding the age groups, it became apparent that *ftxA*-positivity and combined high loads of *F. alocis* were both clearly associated with the older group (>40 years) in the present study population. This suggests that FtxA might contribute to enhancing the survival chances of this bacterium within the periodontal pocket during progression of the disease.

This was also evident when assessing the association of these parameters with the presence of *A. actinomycetemcomitans*. These observations further support the potential use of implementing diagnostic procedures based on the detection of the simultaneous presence of *ftxA*-positive *F. alocis* and *A. actinomycetemcomitans*. Finally, the observation that women in the assessed population exhibited a higher prevalence and higher loads of *F. alocis* compared to men may indicate that hormonal regulation may affect the behavior of this species in the periodontal or oral environment in general (Sharma et al., 2018; Gil-Montoya et al., 2021; Park et al., 2023).

## 5 Conclusions

We conclude that high loads of *F. alocis* and the presence of its *ftxA* gene together are associated with the occurrence and severity of periodontitis. This emphasizes that *F. alocis* and the expression of its *ftxA* gene are associated with the progression of the disease. A further observation of ecological significance is that *ftxA*-positive *F. alocis* strains more frequently co-existed with *A. actinomycetemcomitans*, and an observation of diagnostic potential is that these ecological changes were associated with periodontal tissue destruction (*i.e.*, increased CAL). These findings pave the way for more studies on the pathogenic potential, risk assessment value, and diagnostic utility of *F. alocis* and its FtxA, potentially also based on the simultaneous detection of *ftxA* and *A. actinomycetemcomitans*.

## Data Availability

The datasets presented in this article are available. Requests to access the datasets should be directed to jan.oscarsson@umu.se.
